# Replication stress links Geminin depletion to centrosome amplification

**DOI:** 10.64898/2026.06.30.735730

**Published:** 2026-07-02

**Authors:** Inês B. Santos, David M. Glover

**Affiliations:** 1 Division of Biology and Biological Engineering, California Institute of Technology, Pasadena, CA, USA

**Keywords:** DNA damage, replication stress, Geminin, centriole disengagement, centrosome duplication, cell cycle

## Abstract

The timing of DNA replication and centrosome duplication is tightly regulated with cell cycle progression to ensure the faithful duplication of the genome during cell division. Both DNA and centrosomes are licensed for replication in late telophase/early G1, replicated in S phase and segregated during mitosis; yet how defects in DNA replication licensing are coupled to centrosome homeostasis remains poorly understood. Here, we show that depletion of the replication licensing inhibitor Geminin in proliferating mouse embryonic fibroblasts induces robust centrosome amplification together with impaired primary cilium assembly. Rather than promoting whole-genome reduplication, knockdown of Geminin triggers a replication stress response, characterized by DNA damage accumulation throughout the cycle, and activation of an ATR-dependent DNA damage response. Mechanistically, Geminin depletion-induced replication stress activates the ATR-Chk1-Wee1 checkpoint axis prolonging G2 and leading to premature centriole disengagement and centrosome amplification. These findings identify replication stress as the signaling module that couples defective DNA replication licensing to centrosome amplification.

## Results and discussion

Accurate duplication of both genome and the centrosome is essential to preserve genomic integrity. Each centrosome consists of two barrel-shaped, orthogonal oriented centrioles, surrounded by a protein-rich pericentriolar matrix (PCM). The centriole duplication cycle starts with centriole disengagement during the telophase of the previous cell cycle. Centriole disengagement is under the control of mitotic kinase PLK1 and the protease Separase [1][2][3]. As centrioles disengage, they lose their orthogonal orientation and steric hindrance for procentriole formation, thus licensing them for duplication during the next S-phase. The duplicated centrosomes, each containing a pair of engaged centrioles, separate at mitotic onset to orient the bipolar mitotic spindle.

DNA replication is tightly regulated to occur once per cell cycle. This is ensured by separating DNA replication into two steps. First, in late mitosis/early G1, replication origins are licensed through assembly of a pre-replication complex (preRC). The preRC assembles sequentially through binding of origin recognition complex (Orc) to DNA origins, followed by Cdc6 recruitment, which then recruits the replicative helicase, Mcm2–7, and the licensing factor Cdt1. The second step occurs during S phase, when replication forks are fired at licensed origins. Multiple mechanisms exist to control Cdt1 protein levels: ubiquitin mediated-degradation of Cdt1 blocks DNA licensing in S phase; phosphorylation of Cdt1 by Cdk1/cyclin A blocks Mcm2–7 recruitment in G2 phase; and the binding of Cdt1 to the inhibitory protein Geminin in S and G2 phases blocks DNA licensing in metazoans [4].

In their initial identification of Geminin, McGarry & Kirschner mapped a 9 amino-acid (aa) destruction box consensus in the N-terminus of Geminin, recognized by mitotic anaphase-promoting complex (APC) which controls Geminin levels. At the G1/S transition, the APC is inactivated and Geminin begins to accumulate, preventing licensing of new origins. Because Geminin has no effect on elongation, DNA replication continues to completion. At the metaphase-anaphase transition, the APC is activated, mediating ubiquitination and the abrupt degradation of Geminin, thereby permitting another round of replication to initiate in the following cell cycle [5].

These processes are coordinated through cell-cycle dependent mechanisms that prevent re-licensing of DNA replication or centriole duplication. Failure to maintain such mechanisms results in replication stress, genomic instability and centrosome amplification, common features of human cancers and developmental disorders [6][7]. In fact, inherited mutations in Geminin and other factors associated with the initial steps of DNA replication fork assembly result in Meier-Gorlin Syndrome (MGS), a rare genetic disorder conferring microcephaly and growth defects.

Loss of Geminin has been reported to induce DNA re-replication and centrosome amplification in multiple experimental systems, yet the mechanistic relationship between these phenotypes remains unclear. In particular, it has not been resolved how centrosome amplification arises under such conditions. Here, we show that depletion of Geminin in proliferating mouse embryonic fibroblasts (MEFs) induces robust centrosome amplification without whole-genome duplication. Instead, Geminin loss triggers a replication stress state that activates a checkpoint response dependent on ATR (Ataxia-Telangiectasia Mutated and RAD3-related) that promotes centriole amplification through a Wee1-Plk1 (Polo-like kinase 1) dependent pathway. Our findings identify replications stress as the signal coupling defective DNA licensing to centrosome amplification and provide a mechanistic framework linking genome surveillance pathways to centrosome duplication mechanisms.

**Figure 1.**
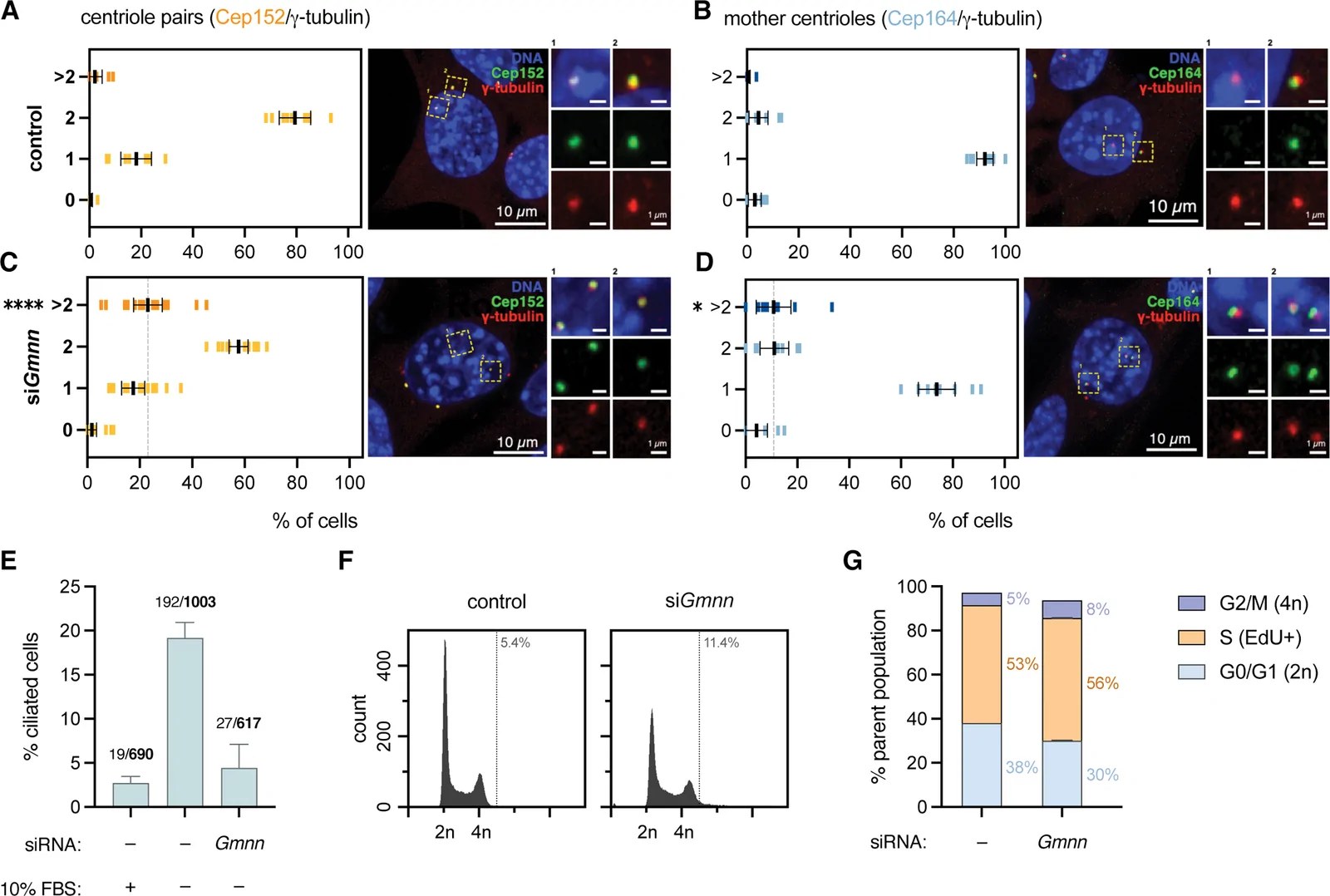
Geminin depletion induces robust centrosome amplification and loss of primary cilia in proliferating mouse embryonic fibroblasts. (A) Graphic representation of the percentage of cells showing 0, 1, 2 and more than 2 (>2) centriole pairs, per cell. Mouse embryonic fibroblasts (MEFs) were stained to reveal Cep152 (green), **γ**-tubulin (red), α-tubulin (not shown) and DNA (blue). Colocalization of Cep152 with **γ**-tubulin identifies centriole pairs/centrosomes. (B) Graphic representation of the percentage of cells showing 0, 1, 2 and more than 2 (>2) mother centrioles, per cell. Mouse embryonic fibroblasts (MEFs) are stained to reveal Cep164 (green), **γ**-tubulin (red), αtubulin (not shown) and DNA (blue). Colocalization of Cep164 with **γ**-tubulin identifies mother centrioles. (C) As in (A). MEFs were subjected to Geminin siRNA, and the number of centriole pairs/centrosomes was analyzed 3 days post transfection (dpt). (D) As in (B). MEFs were subjected to Geminin siRNA, and mother centriole number was analyzed 3 dpt. (E) Control or Geminin-depleted MEFs were serum starved for 24 hours and processed to analyze primary cilia assembly using anti-acetylated tubulin and anti-Arl13B antibodies. The percentage of ciliated cells was measured for each group. Number of ciliated cells/total numbers of cells analyzed are depicted above each bar. (F) Control or Geminin-depleted MEFs were analyzed by FACS at 3-dpt. FACS profile from a representative experiment and the percentage of cells with DNA content > 4n are shown. (G) FACS profiles described in (F) were used to quantify the percentage of cells in each cell cycle phase. EdU incorporation was used to determine cells actively replicating DNA (S-phase), and DNA counts were used to identify 2n (G0/G1 cells) and 4n (G2/M) peaks. The distribution of cells in each cell cycle phase is depicted as a percentage of the parental population next to each bar. Data information: In (A-D) the number of centrioles was quantified for each category, and percentile values are shown as vertical bars. Experiments were performed at least 3 times independently. Error bars correspond to mean ± 95% confidence interval (CI). The statistical significance was calculated using the Kruskal-Wallis test, followed by Dunn’s multiple comparisons test for multiple pairwise comparisons. P-values are represented with one (P<0.05), or four (P<0.0001) asterisks. Non-statistical significance is labeled ns. Scale bar, 10 μm; inset scale bar, 1 μm. In (E and H) the average and standard deviation (SD) of three independent experiments are plotted.

To investigate the function of Geminin in cell cycle progression, we examined the effects of inhibiting its expression using short interfering RNA (siRNA) in mouse embryonic fibroblasts (MEFs). Centrosome number was assessed by co-staining to reveal the pan-centriolar marker CEP152 and **γ**tubulin to count the total number of centriole pairs, or by co-staining to reveal the distal appendage protein CEP164 and **γ**tubulin to quantify mother centrioles. Control MEFs predominantly contained one or two centrosomes, reflecting normal cell cycle progression. In contrast, Geminin-depleted cells exhibited increased numbers of total centrosomes that were positive for Cep152 (2.14±1.20% in control vs. 23.09±2.59% in Geminin-depleted cells, Figure 1A/C), corresponding to an increase in cells that had greater than two Cep164 positive mother centrioles (0.37±0.22% in control vs. 10.82±2.98% in Geminin-depleted cells, Figure 1B/D). Efficient depletion of Geminin was confirmed both at the transcript and at the protein level, and four independent siRNA oligonucleotides produced comparable centrosome amplification phenotypes, demonstrating that the observed defects were not attributable to off-target effects (Supplemental Figure 1).

Because centrioles also template basal body formation necessary for primary cilium assembly, we next examined whether Geminin depletion compromised this centrosome associated function. Following serum starvation, Geminin-depleted MEFs exhibited a pronounced decrease in primary cilia assembly compared with serum-starved control cells (4.43±1.53% in Geminin-depleted vs. 19.17±1.01% in control cells, Figure 1E), indicating that centrosome amplification was accompanied by an impaired ability of mother centrioles to generate competent basal bodies.

Geminin depletion has previously been proposed to induce centrosome amplification as a consequence of DNA re-replication. To determine if the observed phenotype reflected whole-genome duplication, we analyzed DNA content and thereby, cell cycle distribution by flow cytometry. Geminin depletion resulted in a small but consistent accumulation of cells in S/G2, consistent with impaired DNA replication, but only a modest increase in cells with DNA content above 4n (11.4% vs. 5.4%, Figure 1F/G). Importantly, no evidence of a distinct polyploid population was observed, merely a widening of the 4n tail. These observations suggest that in MEFs, Geminin depletion perturbs DNA replication without re-duplicating the genome, raising the possibility that replication stress, rather than polyploidization or cytokinesis failure, underlies centrosome amplification.

**Figure 2.**
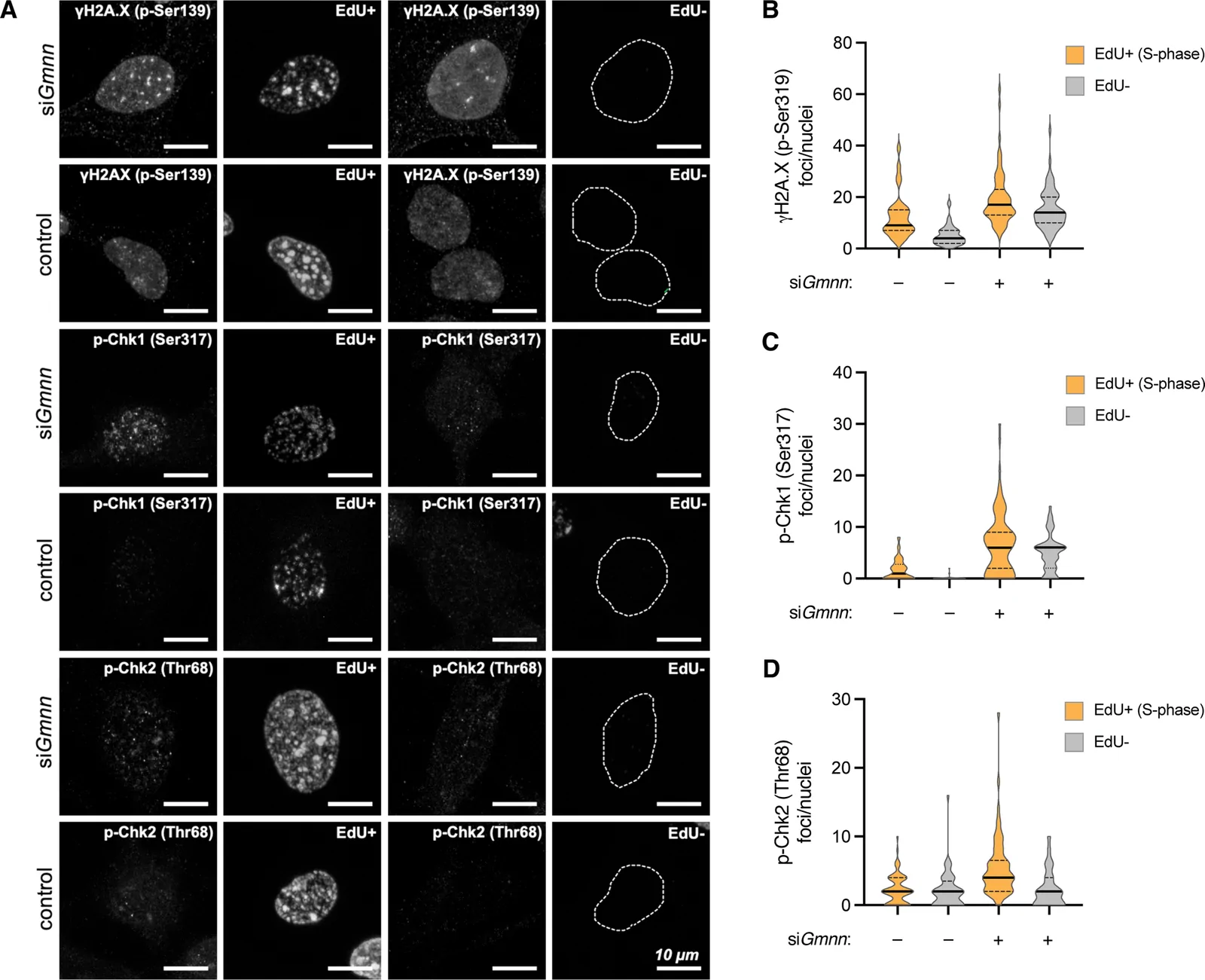
Geminin depletion induces a replication-stress associated DNA damage response without whole genome-duplication. (A) Control and Geminin-depleted MEFs were treated with pulse-labelled EdU for 1 hour prior to fixation, to identify cells actively replicating DNA. Click-it reaction and immunostaining against **γ**H2A.x (phospho-Ser139) or Chk1 (phospho-Ser317) or Chk2 (phospho-Thr68) was carried out as described in the Materials and Methods. Representative images for each antibody are shown. Scale bar, 10 μm. (B) The number of **γ**H2A.x (phospho-Ser139) foci per nuclei for at least 150 cells per condition was quantified using FIJI software and plotted. (C) The number of Chk1 (phospho-Ser317) foci per nuclei for at least 150 cells per condition was quantified using FIJI software and plotted. (D) The number of Chk2 (phospho-Thr68) foci per nuclei for at least 150 cells per condition was quantified using FIJI software and plotted. Data information: Representative images are shown in (A). In (B-D) at least 200 cells per condition were quantified in 3-independent experiments. For each data set, black line represents median and dashed lines represent upper and lower quartiles.

To distinguish between the above possibilities, we examined activation of the DNA damage response during S phase by pulse-labelling cells with EdU (5-ethynyl-2′-deoxyuridine) and quantifying markers of DNA damage signaling. We found that Geminin-depleted cells exhibited an increase in γH2AX foci relative to control cells, indicating accumulation of DNA lesions during DNA replication (Figure 2A/B). This response was accompanied by a significant increase in phosphorylated Chk1 foci, in line with activation of the ATR-dependent replication stress checkpoint (Figure 2A/C).

Interestingly, increased numbers of both γH2AX and phosphorylated Chk1 nuclear foci were present in cells that did not show EdU incorporation, supporting the notion that Geminin-depleted MEFs are in a replication stress state and that some origin licensing is likely occurring outside of G1. In contrast, phosphorylation of Chk2 remained largely unchanged following Geminin depletion, with a slight increase in nuclear foci detected in S phase cells (Figure 2A/D). This is likely caused by replication fork stalling collapsing into DNA double strand breaks (DSB) and activating the DNA damage response kinase ATM (Ataxia-Telangiectasia Mutated) and its downstream effector Chk2. Taken together, these findings indicate that Geminin depletion preferentially activates an ATR-mediated replication stress response rather than a DNA double-strand break signaling pathway. Activation of the ATR/Chk1 axis is known to suppress the expression of a mitotic gene network during DNA replication to prevent mitotic entry with under-replicated DNA [8][9]. Together with the cell cycle analysis, these observations argue against extensive genome re-replication as the primary consequence of Geminin knockdown in MEFs. Instead, our findings support a model in which replication licensing defects generate replication stress, leading to activation of ATR–Chk1 signaling before the onset of centrosome amplification.

**Figure 3.**
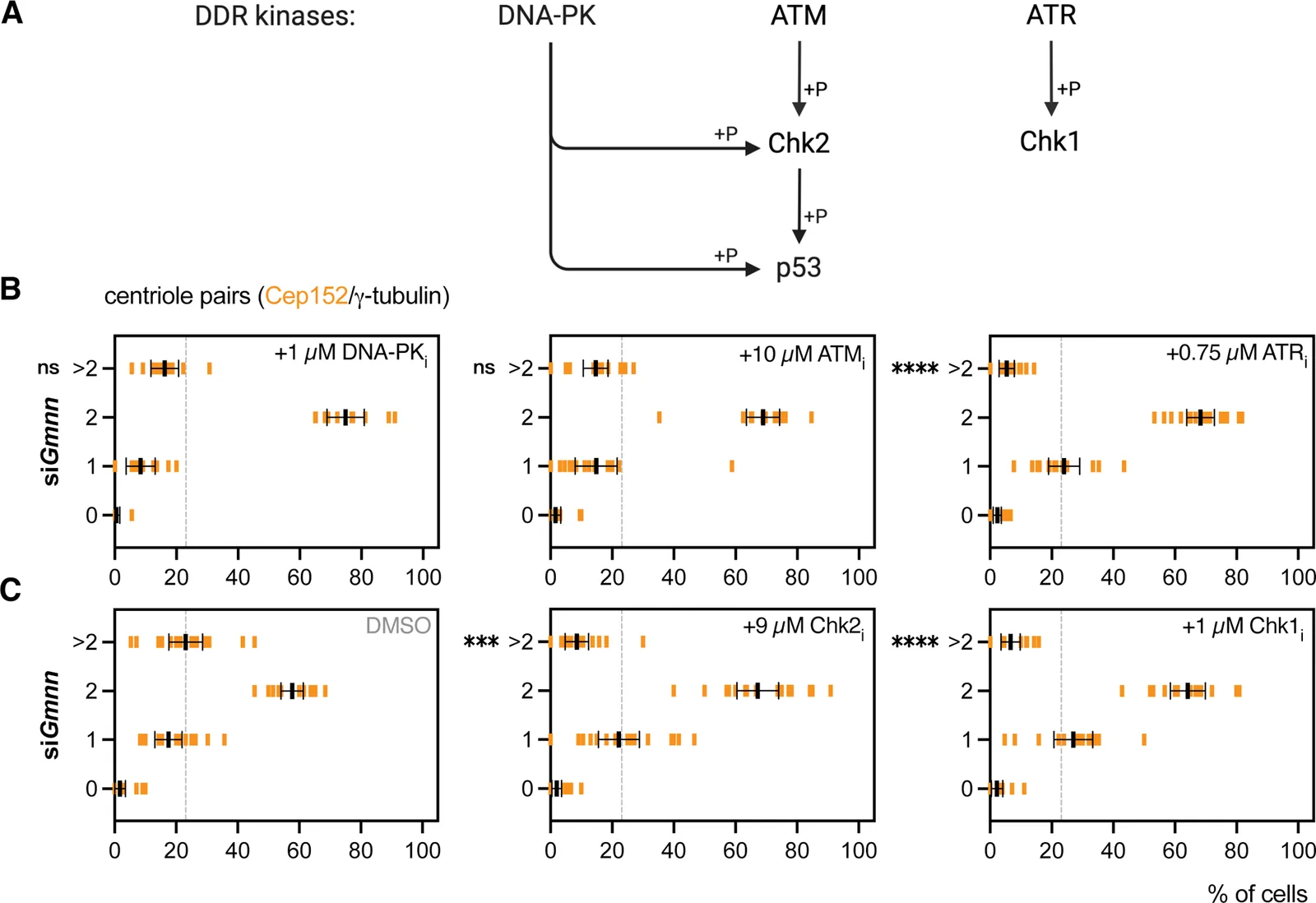
Checkpoint kinase signaling downstream of ATR is required for Geminin-depletion induced centriole amplification. (A) Schematic representation of the DNA damage response molecular pathway. Created in BioRender. Santos, IB. (2026) https://BioRender.com/dz42sw8. (B-C) Graphic representation of the percentage of cells showing 0, 1, 2 and more than 2 (>2) centriole pairs, per cell. Geminin-depleted MEFs were stained to reveal Cep152, **γ**-tubulin, α-tubulin and DNA. Colocalization of Cep152 with **γ**-tubulin identifies centriole pairs/centrosomes. (B) Geminin-depleted MEFs were individually treated with inhibitors against the DNA damage response kinases DNA-PK, ATM or ATR. (C) Geminin-depleted MEFs were individually treated with inhibitors of the checkpoint kinases Chk1, Chk2 or vehicle control (DMSO). Data information: In (B-C) cells were transfected with siRNA against Geminin; cells were fixed and centriole number was analyzed at 3-dpt. The number of centrioles was quantified for each category, and percentile values are shown as vertical bars. Experiments were performed at least 3 times independently. Error bars correspond to mean ± 95% confidence interval (CI). The statistical significance was calculated using the Kruskal-Wallis test, followed by Dunn’s multiple comparisons test for multiple pairwise comparisons. P-values are represented with three (P<0.001) or four (P<0.0001) asterisks. Non-statistical significance is labeled ns. Inhibitors were added to cells in culture, at 1-dpt (inhibitor treatment time: 2 days prior to fixation).

We next asked whether activation of the ATR-Chk1 checkpoint contributes functionally to centrosome amplification. To this end, we examined the requirement of DNA damage response kinases for the centrosome amplification process (Figure 3A). Geminin-depleted MEFs were treated with selective inhibitors of DNA-PK (DNA-dependent protein kinase), ATM or ATR and centriole amplification was quantified. Inhibition of ATR markedly suppressed Geminin depletion-induced centrosome amplification (ATR_i_: 5.36±1.15% vs. DMSO: 23.09±2.59%, Figure 3B). Neither ATM inhibition, nor DNA-PK inhibition, had any significant effect upon centrosome amplification following Geminin depletion (ATM_i_: 14.64±1.90%; DNA-PK_i_: 16.23±2.01% vs. DMSO: 23.09±2.59%, Figure 3B). These findings indicate that activation of ATR, rather than the canonical DSB response, is specifically required for centriole amplification following replication licensing defects. To initiate DNA repair and prevent mitotic entry in cells having damaged DNA, ATR activates the checkpoint kinase Chk1, while ATM activates Chk2. We next examined the contribution of these downstream effector kinases. Pharmacological inhibition of Chk1 suppressed centrosome amplification in Geminin-depleted cells (Chk1_i_: 6.62±1.44% vs. DMSO: 23.09±2.59%, Figure 3C), whereas inhibition of Chk2 had a smaller but still significant effect in reducing centrosome number (Chk2_i_: 8.54±1.79% vs. DMSO: 23.09±2.59%, Figure 3C). The suppression of centrosome amplification by Chk1 inhibition is in line with a requirement for ATR-dependent DNA damage signaling. Interestingly, knockdown of Geminin in *Drosophila* cells has previously been reported to induce re-replication, that can be rescued by silencing of Chk1 but not Chk2 [10][11]. Together, these findings demonstrate that checkpoint signaling not only marks replication stress but is essential to mediate the centrosome amplification response. The fact that inhibition of Chk2 but not ATM also had an effect in rescuing Geminin-induced centrosome amplification was surprising, but perhaps not completely unexpected. While no functional relationship has been described till now, Mihaylov *et* al. reported to have isolated Geminin during the analysis of Chk2-associated proteins, in HeLa cells. However, the convergence of checkpoint kinase signaling via Chk2 will require further studies to identify how it becomes activated.

**Figure 4.**
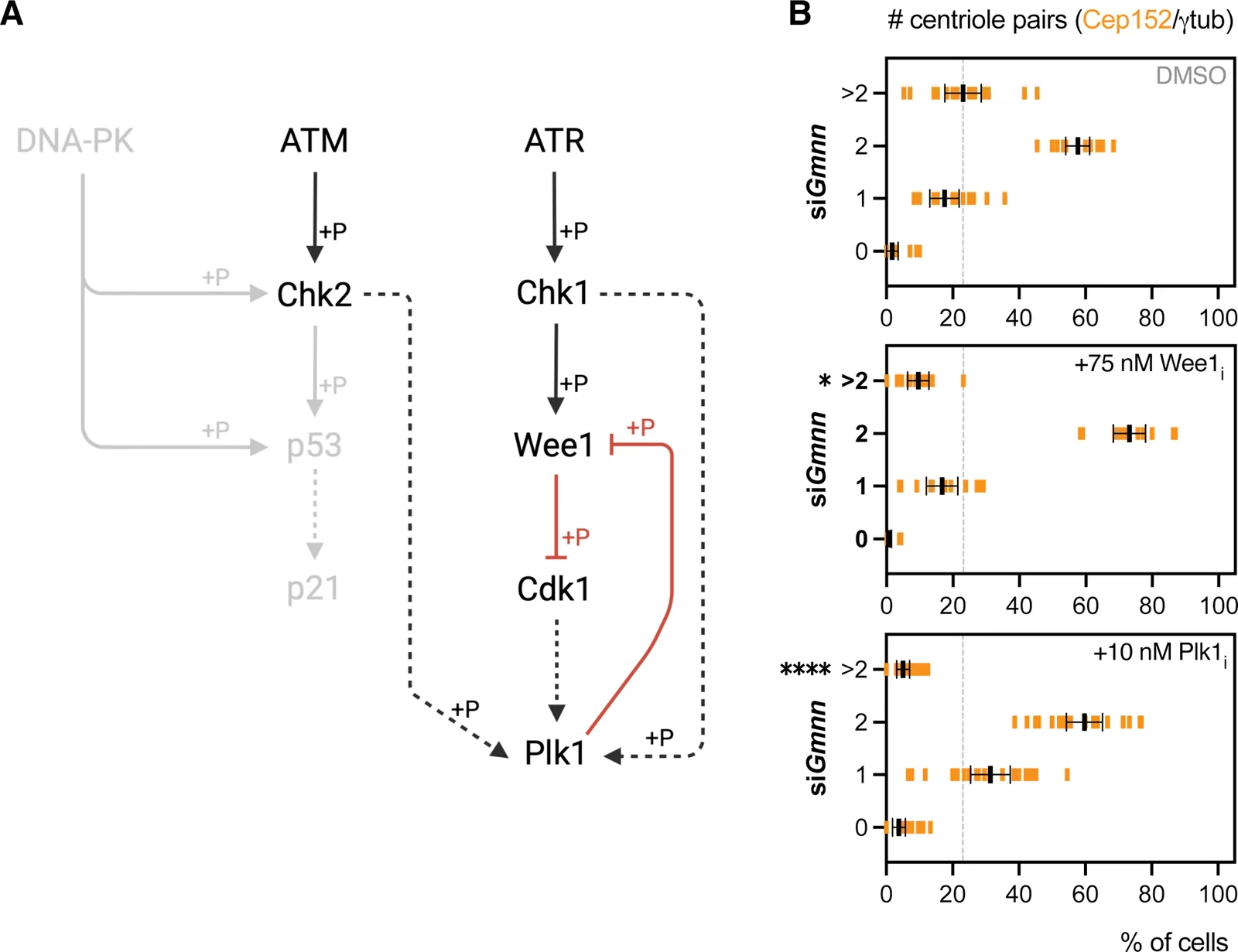
Wee1 and Plk1 define a CDK-dependent mechanism linking replication stress to centriole amplification. (A) Schematic representation of the molecular pathway regulating pre-mature centriole disengagement upon replication stress. Created in BioRender. Santos, IB. (2026) https://BioRender.com/9nwo5s7. (B) Graphic representation of the percentage of cells showing 0, 1, 2 and more than 2 (>2) centriole pairs, per cell. Geminin-depleted MEFs were stained to reveal Cep152, **γ**-tubulin, α-tubulin and DNA. Colocalization of Cep152 with **γ**-tubulin identifies centriole pairs/centrosomes. Geminin-depleted MEFs were individually treated with inhibitors against mitotic kinases Plk1 and Wee1. Data information: In (B) cells were transfected with siRNA against Geminin; cells were fixed and centriole number was analyzed at 3-dpt. The number of centrioles was quantified for each category, and percentile values are shown as vertical bars. Experiments were performed at least 3 times independently. Error bars correspond to mean ± 95% confidence interval (CI). The statistical significance was calculated using the Kruskal-Wallis test, followed by Dunn’s multiple comparisons test for multiple pairwise comparisons. P-values are represented with one (P<0.05), or four (P<0.0001) asterisks. Inhibitors were added to cells in culture at 1-dpt (inhibitor treatment time: 2 days prior to fixation).

ATR–Chk1 signaling coordinates multiple cell-cycle regulators that control entry into mitosis, including the activation Wee1 kinase that phosphorylates CDK1 to prevent mitotic entry and prolong G2 (Figure 4A) [12]. Accordingly, we found that pharmacological inhibition of Wee1 markedly suppressed the accumulation of supernumerary centrioles in Geminin-depleted MEFs (Wee1_i_: 9.54±1.49% vs. DMSO: 23.09±2.59%, Figure 4B), demonstrating its requirement in the ATR checkpoint pathway to inhibit Cdk1. Because Plk1 is a key regulator of centriole disengagement to license centriole duplication, we asked whether its activity was required to mediate centrosome amplification following Geminin depletion. We found that inhibition of Plk1 suppressed the accumulation of supernumerary centrioles in Geminin-depleted MEFs (Plk1_i_: 5.08±0.94% vs. DMSO: 23.09±2.59%, Figure 4B). Together, these findings identify a Wee1–Plk1 signaling module downstream of ATR checkpoint pathway, in the regulation of centrosome homeostasis following replication stress.

Premature centriole disengagement licenses centrioles for duplication independently of the normal cell cycle program and is a known mechanism underlying centriole amplification. In fact, mild replication stress induced by nanomolar doses of aphidicolin has recently been shown to cause premature centriole disengagement in G2, via the ATR-Chk1 axis of the DNA damage repair pathway [13]. These authors demonstrated that mild replication stress resulted in dampening of Plk1 activity, insufficient to promote mitotic entry, but sufficient to support premature centriole disengagement via Separase-mediated cleavage of Pericentrin. Inhibition of ATR in such conditions, rescued Plk1 activity, thus preventing centriole disengagement. Thus, under replication stress conditions, the ATR-Chk1 axis prevents the full activation of Plk1, consistent with studies that show Chk1 acting upstream of and directly phosphorylating Plk1[14]. To determine whether Geminin depletion induces this licensing defect, we examined centriole architecture by expansion microscopy. The increased frequency of disengaged centrioles observed in Geminin depleted cells suggests that disengagement is premature compared with control cells (Supplemental figure S3). Such premature disengagement is consistent with untimely centriole licensing preceding centrosome amplification.

Together, our findings support a model in which loss of Geminin leads to defective DNA replication licensing and induces replication stress that activates ATR/Chk1 checkpoint signaling, leading to Wee1/Plk1-dependent premature centriole disengagement and subsequent centrosome amplification (Figure 4A).

Faithful duplication of the genome and centrosome depends on the coordinated licensing of both DNA replication origins and centriole duplication. Although Geminin has long been recognized as a key inhibitor of DNA replication licensing, how its loss promotes centrosome amplification has been unresolved. Our study demonstrates that, in proliferating MEFs, Geminin depletion induces centrosome amplification not through extensive genome re-replication, but through activation of a replication stress response. We identify ATR-dependent checkpoint signaling as the molecular link between defective DNA replication licensing and centriole amplification and show that this pathway requires downstream regulation of the Wee1/Plk1 axis to promote premature centriole disengagement.

Centrosome amplification following Geminin depletion has generally been interpreted as a secondary consequence of DNA re-replication or polyploidization. While such outcomes have been reported in transformed human cell lines and other experimental systems, our results indicate that this is not the predominant response in embryonic fibroblasts. Instead, Geminin depletion results in a modest accumulation of cells in S/G2 together with robust activation of γH2AX and phospho-Chk1, but without the emergence of a substantial polyploid population. This distinction has important implications because it uncouples centrosome amplification from changes in DNA content and instead identifies checkpoint signaling itself as the initiating event.

Our data demonstrates that ATR and Chk1 are required for Geminin depletion-induced centrosome amplification, establishing that replication stress signaling, rather than being concurrent, actively drives this phenotype. Previous studies have reported physical association between Geminin and Chk2, raising the possibility that crosstalk between checkpoint pathways contributes to centrosome homeostasis. Whether this reflects convergence between ATR- and ATM-dependent signaling or an independent role for Chk2 in centrosome biology remains to be determined.

Mechanistically, our data position a Wee1–Plk1 module downstream of ATR–Chk1 in regulating centriole licensing. Plk1 is well established as the regulator of centriole disengagement during mitotic exit, yet recent work has suggested that replication stress can also modulate Plk1 activity to induce premature centriole disengagement during G2. Our observations are in accord with this model. Together, these findings support a model in which replication stress signaling lowers the threshold for the licensing of centriole duplication, allowing disengaged centrioles to reduplicate independently of normal cell-cycle progression.

Replication stress is increasingly recognized as a defining feature of oncogene activation, of inherited defects in DNA replication, and of numerous developmental disorders. Centrosome amplification is likewise common in these conditions, although the mechanistic relationship between the two has remained poorly understood. Our findings suggest that centrosome amplification need not arise as a passive consequence of genome instability but may instead represent an active cellular response to replication stress mediated by checkpoint signaling.

In summary, we propose a model in which loss of Geminin leads to defective DNA replication licensing that activates an ATR/Chk1-dependent replication stress response that signals through the inhibitory effects of Wee1 while permitting Plk1 to promote premature centriole disengagement and thereby, centrosome amplification. More broadly, our study identifies replication stress as a signaling mechanism coordinating DNA replication licensing with centrosome duplication, providing a conceptual framework linking genome surveillance pathways to centrosome homeostasis.

## Material and Methods

### Cell culture and transfection.

Mouse embryonic fibroblasts were prepared as described previously [15], and cultured in Dulbecco's Modified Eagle Medium (DMEM) supplemented with 10% fetal bovine serum (FBS). To knockdown the expression of each gene, reverse transfection of siRNA into MEFs was performed in a 24-well dish. The transfection mixture comprised 6 pmol siRNA duplex and 1 μl Lipofectamine RNAiMAX (Invitrogen #13778075) in 100 μl Opti-MEM (Gibco #31985070), per well. Liposomes were gently mixed, added to each well, and incubated for 20 minutes at room temperature. MEFs were resuspended to 0.5×10^5^ cells/mL in DMEM, and 500 μl of cell suspension was added per well and incubated with the transfection mix. siRNA used are listed in Table 1. Inhibitors (or DMSO control) were added with 600 μl of fresh culture media 1 day post-transfection (dpt). Inhibitors and concentrations are listed in Table 2. Cell proliferation, centriole analysis and cell-cycle distribution were assayed at 3-dpt. DNA damage accumulation was assayed 36 hours post-transfection.

### Western-blot.

Cells were harvested and lysed in RIPA buffer containing 1 U/ml Pierce^™^ Universal Nuclease (Thermo Scientific #88700) and protease inhibitors (Roche #04693116001) and incubated on ice for 1 hour. The supernatant fraction was mixed with 4x Laemmli buffer (Bio-Rad #1610747). Equal protein amounts were subjected to electrophoresis on 15% Tris-Glycine Acrylamide gels, transferred to nitrocellulose membranes and immunoblotted with the indicated primary and secondary antibodies. Blots were developed with enhanced chemiluminescence (BioRad #1705061). Primary antibodies are listed in Table 3.

### Fluorescent-activated cell sorting (FACS).

FACS analysis for DNA content was performed using standard procedures and 2 μg/mL DAPI staining. For EdU staining, cells were pulsed with 10 μM EdU for 2 hours, fixed, and processed according to the manufacturer’s protocol (Invitrogen #C10424). Flow cytometry was performed using the Cytoflex S Analyzer (Beckman Coulter) and data were analyzed using CytExpert analysis software. To calculate the percentage of G0/G1, S (EdU+), and G2/M phases, and aneuploid cells (DNA content >4n) cells, cell doublets and sub-G1 apoptotic cells were excluded.

### Immunofluorescence.

Cells were grown on ⌀ 12 mm coverslips, washed with pre-warmed PBS for 3 minutes, and then fixed in ice-cold methanol for 12 minutes at −20 °C, or fixed with 4% formaldehyde for 10 minutes at room temperature. Cells were washed three times, permeabilized in PBS/0.5% Triton X-100 for 15 minutes followed by 3 washes. Blocking was performed in PBS/0.1% Tween-20/10% FBS for 30 minutes at room temperature, followed by overnight incubation with primary antibodies diluted in PBS/0.1% Tween-20/1% FBS, at 4 °C. Cells were washed three times before overnight incubation with secondary antibodies diluted in PBS/0.1% Tween-20/1% FBS, at 4 °C. DNA staining was performed with 0.5 μg/mL DAPI and cells were washed three times before mounting in Vectashield mounting medium (Vector Labs #H-1900–2). Primary and secondary antibodies are listed in Table 3.

### Confocal microscopy.

Slides were imaged on a Leica SP8 Stellaris inverted microscope with 63×/1.4 N.A. oil immersion objective using the Leica Application Suite X software (LAS-X). Images were deconvolved with Huygens Professional version 18.10 (Scientific Volume Imaging, The Netherlands). Images were processed and analyzed with ImageJ version 1.54p. All images shown are the maximum intensity projections of all Z optical sections.

### Quantification and statistical analysis.

A minimum of three biological replicates were performed for each experiment. For centriole quantification, each vertical bar in the graph represents the average of all the cells at that stage from a single field of view, typically 20–30 cells, with at least 200 cells quantified, per condition. Data were analyzed using nonparametric statistical methods. Differences across multiple groups were evaluated with the Kruskal–Wallis test followed by Dunn’s multiple comparisons test for multiple pairwise comparisons. For nuclear foci quantification, ImageJ was used to segment isolated nuclei and quantify the number of foci within each. Nuclei were then scored for positive or negative EdU incorporation, to distinguish S phase cells. Statistical tests are indicated in figure legends. All data was plotted and analyzed using GraphPad Prism 11.0.2.

## Supplementary Material

Supplement 1

## Figures and Tables

**Table 1.** siRNAs used in this work

Target gene	Target sequence	Supplier	Gene ID
Geminin-1	CAGGAATTTGATTCTGAAGAA	QIAGEN	SI01053843
Geminin-2	CAGGAAGCCTTTGATCTTATA	QIAGEN	SI01053850
Geminin-3	CCGCCTGAGAAAGGAGAATAA	QIAGEN	SI01053857
Geminin-4	ATCGAGAGGCTGAGTAATGAA	QIAGEN	SI01053864

**Table 2.** Inhibitors used in this work.

Target	Inhibitor	Concentration	Supplier	Catalog No.
DNA-PK	KU-57788	1 μM	MCE	HY-11006
ATM	KU-55933	10 μM	Selleck	S1092
ATR	ETR-46464	0.75 μM	Selleck	S8050
Chk1	PF-477736	1 μM	Selleck	S2904
Chk2	BML-277	9 μM	Selleck	S8632
Wee1	MK-1775	75 nM	Selleck	S1525
Plk1	BI-2536	10 nM	Selleck	S1109
Plk4	LCR-263	150 nM	Selleck	S7837

**Table 3.** Antibodies used in this work.

Antibody	Raised in	Supplier	Reference
Arl13B	Rabbit	Proteintech	17711-1-AP
Cep152	Rabbit	Abcam	ab183911
Cep164	Rabbit	Proteintech	22227-1-AP
Chk1 (p-Ser317)	Rabbit	Cell Signaling Technology	12302
Chk2 (p-Thr68)	Rabbit	Cell Signaling Technology	2661
GAPDH	Rabbit	Abcam	ab9485
Geminin	Rabbit	Proteintech	10802-1-AP
GTU88 (**γ**-tubuiin)	Mouse	Sigma	T6557
α-tubulin	Guinea pig	ABCD antibodies	AA345
acetylated tubulin	Mouse	Sigma	T7451
**γ**H2A.X (p-Ser139)	Rabbit	Abcam	ab81299
