# Supplementary material for: Replication stress links Geminin depletion to centrosome amplification
Source: bioRxiv. 2026 Jul 2:2026.06.30.735730. Preprint. [Version 2] doi: 10.64898/2026.06.30.735730 (PMC13536914; doi:10.64898/2026.06.30.735730)

bioRxiv preprint doi: https://doi.org/10.64898/2026.06.30.735730; this version posted July 2, 2026. The copyright holder for this preprint (which was not certified by peer review) is the author/funder, who has granted bioRxiv a license to display the preprint in perpetuity. It is made available under a CC-BY 4.0 International license.

Supplementary Figure 1. Validation of Geminin-depletion and individual siRNA phenotypes. (A) Graphic representation of the percentage of cells showing more than 2 (>2) centriole pairs, per cell (centrosome amplification phenotype). Mouse embryonic

bioRxiv preprint doi: https://doi.org/10.64898/2026.06.30.735730; this version posted July 2, 2026. The copyright holder for this preprint (which was not certified by peer review) is the author/funder, who has granted bioRxiv a license to display the preprint in perpetuity. It is made available under a CC-BY 4.0 International license.

fibroblasts (MEFs) were stained to reveal Cep152, γ-tubulin, α-tubulin and DNA. Colocalization of Cep152 with γ-tubulin identifies centriole pairs/centrosomes. 4 siRNA oligonucleotides against Geminin were tested for their efficacy to induce mother centriole amplification in MEFs. (B) Graphic representation of the percentage of cells showing more than 2 (>2) mother centrioles, per cell (centrosome amplification phenotype). Mouse embryonic fibroblasts (MEFs) are stained to reveal Cep164, γ-tubulin, α-tubulin and DNA. Colocalization of Cep164 with γ-tubulin identifies mother centrioles. 4 siRNA oligonucleotides against Geminin were tested for their efficacy to induce centriole/centrosome amplification in MEFs. (C) RT-qPCR analysis of relative levels of Geminin mRNA transcripts in MEFs treated with siRNA against Geminin at the indicated timepoints. Average from two biological samples, three technical replicates within each. (D) Western-blot analysis of Geminin protein levels in MEFs treated with individual siRNAs against Geminin. 4 siRNA oligonucleotides against Geminin were tested for their efficacy to deplete endogenous Geminin.

Supplementary Figure 2. Plk4 inhibitor validates centriole quantification assays. Graphic representation of the percentage of cells showing 0, 1, 2 and more than 2 (>2) centriole pairs, per cell. Control and Geminin-depleted MEFs were stained to reveal Cep152, γ-tubulin, α-tubulin and DNA. Colocalization of Cep152 with γ-tubulin identifies centriole pairs/centrosomes. MEFs were treated with the Plk4 inhibitor, centrinone. The number of centrioles was quantified for each category, and percentage values are shown as vertical bars. Experiments were performed at least 3 times independently. Error bars correspond to mean ± 95% confidence interval (CI). $Plk4_i$ was added to cells in culture at 1-dpt (inhibitor treatment time: 2 days prior to fixation).

Supplemental Figure 3. Premature centriole disengagement in Geminin-depleted cells. Expansion microscopy (ExM) images of centrioles in control and Geminin-depleted MEFs stained against acetylated-tubulin and Cep164 (not shown).

Supplementary Figure 1

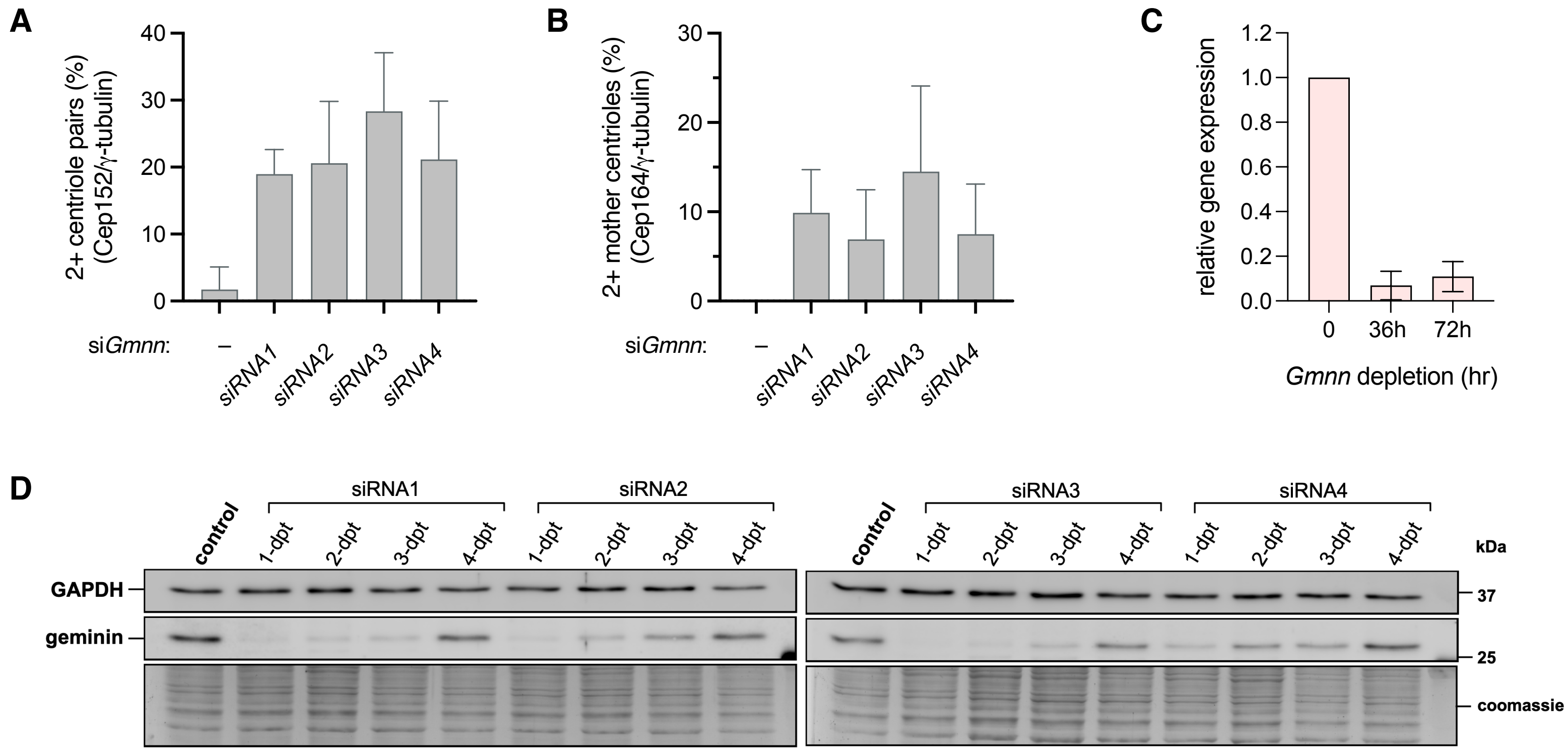

Supplementary Figure 2

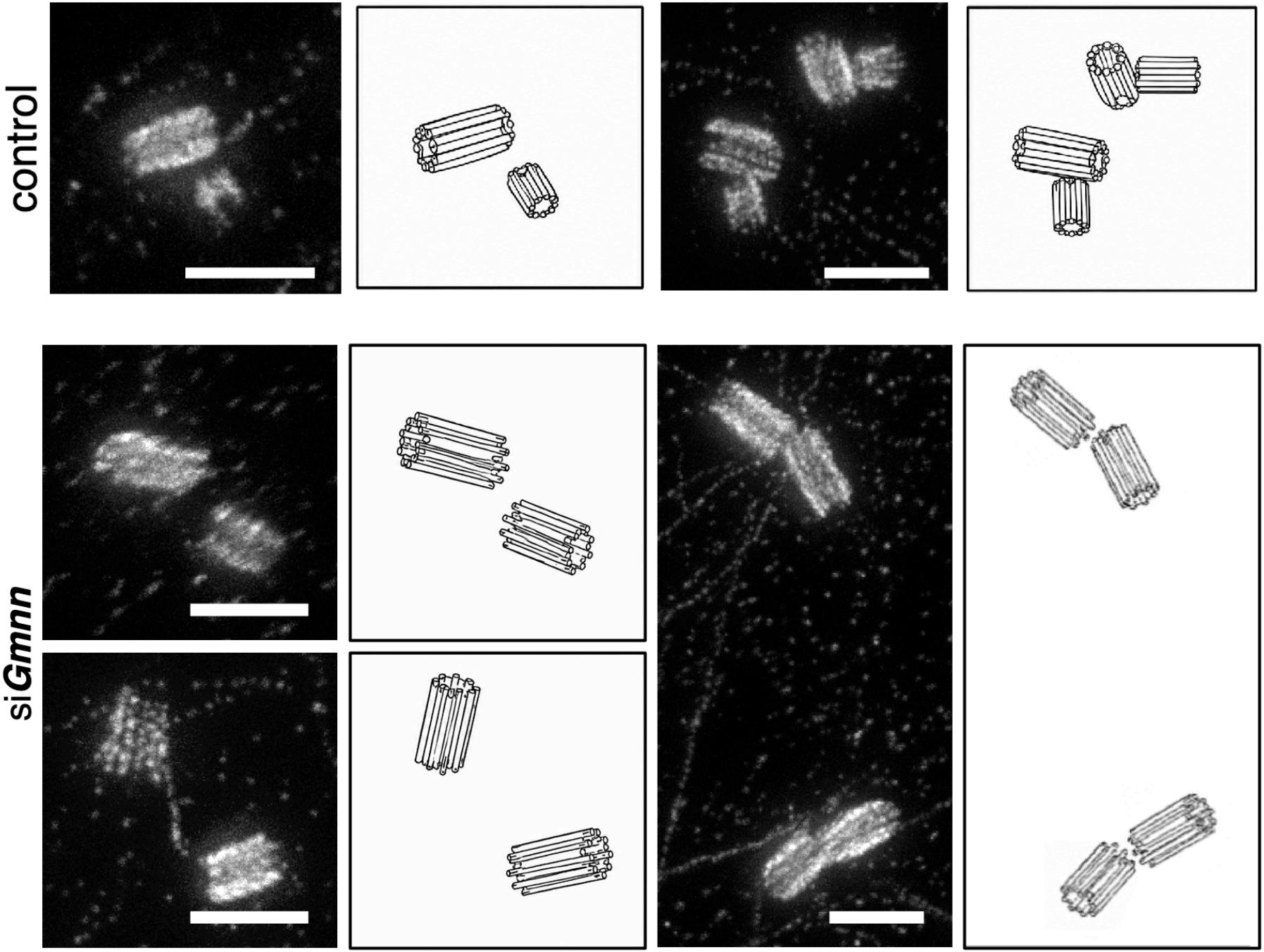

Supplementary Figure 3

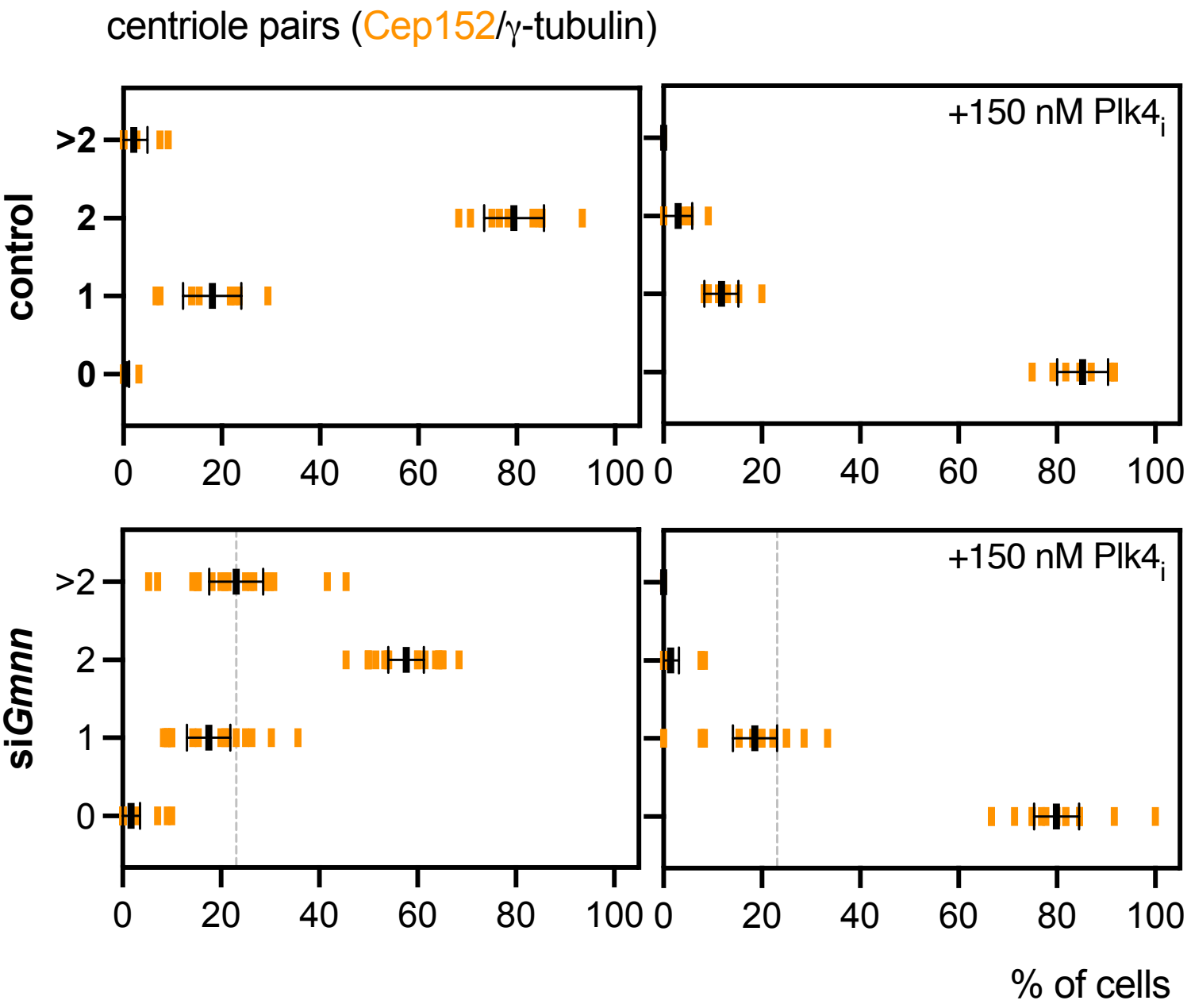

Supplement: Supplementary file 1 [file NIHPP2026.06.30.735730v2-supplement-1.pdf]
